# Enhancing the Impact of Chemotherapy on Ewing Sarcoma Cells through Combination with Cold Physical Plasma

**DOI:** 10.3390/ijms24108669

**Published:** 2023-05-12

**Authors:** Andreas Nitsch, Sara Qarqash, Sarah Römer, Janosch Schoon, Axel Ekkernkamp, Maya Niethard, Johannes C. Reichert, Georgi I. Wassilew, Mladen V. Tzvetkov, Lyubomir Haralambiev

**Affiliations:** 1 Center for Orthopedics, Trauma Surgery and Rehabilitation Medicine, University Medicine Greifswald, Ferdinand-Sauerbruch-Straße, 17475 Greifswald, Germany; andreas.nitsch@med.uni-greifswald.de (A.N.); sara.qarqash@med.uni-greifswald.de (S.Q.); janosch.schoon@med.uni-greifswald.de (J.S.); ekkernkamp@ukb.de (A.E.); maya.niethard@helios-gesundheit.de (M.N.); johannes.reichert@med.uni-greifswald.de (J.C.R.); georgi.wassilew@med.uni-greifswald.de (G.I.W.); 2 Department of General Pharmacology, Institute of Pharmacology, Center of Drug Absorption and Transport (C_DAT), University Medicine Greifswald, 17487 Greifswald, Germany; sarah.roemer@med.uni-greifswald.de (S.R.); mladen.tzvetkov@uni-greifswald.de (M.V.T.); 3 Department of Trauma and Orthopaedic Surgery, BG Klinikum Unfallkrankenhaus Berlin, Warener Straße 7, 12683 Berlin, Germany; 4 Sarcoma Centre, HELIOS-Klinikum Berlin-Buch, Schwanebecker Chaussee 50, 13125 Berlin, Germany

**Keywords:** Ewing sarcoma, chemotherapy, cold physical plasma, doxorubicin, vincristine

## Abstract

Although Ewing’s sarcoma (ES) is a rare, but very aggressive tumor disease affecting the musculoskeletal system, especially in children, it is very aggressive and difficult to treat. Although medical advances and the establishment of chemotherapy represent a turning point in the treatment of ES, resistance to chemotherapy, and its side effects, continue to be problems. New treatment methods such as the application of cold physical plasma (CPP) are considered potential supporting tools since CPP is an exogenous source of reactive oxygen and nitrogen species, which have similar mechanisms of action in the tumor cells as chemotherapy. This study aims to investigate the synergistic effects of CPP and commonly used cytostatic chemotherapeutics on ES cells. The chemotherapy drugs doxorubicin and vincristine, the most commonly used in the treatment of ES, were applied to two different ES cell lines (RD-ES and A673) and their IC_20_ and IC_50_ were determined. In addition, individual chemotherapeutics in combination with CPP were applied to the ES cells and the effects on cell growth, cell viability, and apoptosis processes were examined. A single CPP treatment resulted in the dose-dependent growth inhibition of ES cells. The combination of different cytostatics and CPP led to significant growth inhibition, a reduction in cell viability, and higher rates of apoptosis compared to cells not additionally exposed to CPP. The combination of CPP treatment and the application of cytostatic drugs to ES cells showed promising results, significantly enhancing the cytotoxic effects of chemotherapeutic agents. These preclinical in vitro data indicate that the use of CPP can enhance the efficacy of common cytostatic chemotherapeutics, and thus support the translation of CPP as an anti-tumor therapy in clinical routine.

## 1. Introduction

Ewing’s sarcoma (ES) is the second-most-common bone tumor in humans and yet, with an annual incidence of up to 1.2 cases/million [1], a rare malignancy [2,3]. However, the incidence varies among different population subgroups, showing strong racial variability, with white Caucasians being most commonly affected by ES [1,4,5,6]. Another typical feature of ES is the onset in childhood and adolescence, with a peak between the age of 5 and 24 years [5,7,8]. Recent epidemiological studies indicate that in some Western European countries and Oceania, incidence rates as high as 9.97 cases per million among 10–19-year-olds can be observed [1]. ES usually develops primarily in bone, although soft tissue manifestations are also possible [9,10,11]. ES tumor development is mediated by a chromosomal translocation between TET (ten eleven translocation) and ETS (Erythroblast Transformation Specific) genes, mostly via reciprocal chromosomal translocation (t(11;22) (q24;q12)), and results in oncogenic EWS/FLI1 gene fusion [12,13]. This fusion protein seems to be one of the important pathogenic transcription factors for ES development [14,15,16]. The prognosis of patients with ES depends on the histological grading, extent of metastasis, tumor localization, and adequate surgical margins of resection [3,17].

Since the establishment of chemotherapy in oncology, the concept of radical surgery has been reconsidered as treatment strategies involving systemic adjuvant therapy combined with less extensive local surgery can achieve similar results [18]. The survival rate of ES patients in the 1960s equaled approximately 10% [19]. The current treatment strategies have improved the prognosis of ES patients markedly [20,21,22,23], reaching a survival rate of approximately 60% [24,25,26,27]. These strategies include various combinations of chemotherapeutics such as doxorubicin (DOX), vincristine (VIN), and ifosfamide (IFO) [28]. The greatest limitations and dangers for the patient are attributed to the unwanted side effects of chemotherapy, most commonly nephrotoxicity [29,30,31], cardiotoxicity [32,33,34], neurotoxicity, and hepatotoxicity [35,36].

The local treatment of ES involves surgical excision in combination with preoperative or postoperative radiotherapy to eliminate possible tumor residues at the margin of resection [26]. Although the overall survival rate of ES patients has improved, the general outcome often remains unsatisfactory, while ES treatment is still demanding [37]. Facing these challenges, the application of cold physical plasma (CPP) as a new treatment method is moving into the focus of oncology. CPP, also referred to as cold atmospheric plasma, is an ionized gas formation [38] containing numerous elements including electrons and ions, electric fields, UV radiation, and reactive oxygen and nitrogen species (ROS). The latter are held responsible for the important biomedical effects of CPP [39]. It is assumed that, due to alterations in metabolism, cancer cells are more susceptible to CPP-mediated extracellular ROS [40,41]. CPP devices are commercially available and have regulatory approval for medical purposes [42,43]. They are mainly used to induce wound healing [44,45,46,47]. In the field of oncology, there are many studies delineating CPP effects [48,49] on numerous types of cancer such as melanoma [50,51], breast cancer [52,53,54], glioblastoma [55,56], colorectal cancer [57,58,59], renal cancer [60], cervical cancer [61,62], leukemia [63], pancreatic cancer [64,65], and head and neck cancer [66]. Previous studies have also reported the effects of CPP on bone tumors. The isolated direct and indirect application of CPP has been investigated on osteosarcoma cells [67,68,69,70], but also on Ewing sarcoma [71] and chondrosarcoma cells [72,73]. Initial evidence exists for the use of CPP to sensitize cancer cells or tissues to existing chemotherapy [74,75]. Therefore, the aim of the present study is to investigate the potential synergistic cytotoxicity of CPP and commonly used cytostatic chemotherapeutics on ES cells, focusing on cell growth, cell viability, and apoptosis-related processes.

## 2. Results

### 2.1. Reduction in Cell Viability and Proliferation Rate by Cytostatics

The effectiveness of the most common cytostatics applied in the clinical treatment of ES—DOX and VIN—was examined. For this purpose, the cell lines A673 and RD-ES were incubated in media containing different concentrations (DOX: 10^−11^ to 10^−4^ M, VIN: 10^−12^ to 10^−5^ M) of the cytostatics and cell viability was examined after 72 h incubation.

Our investigations showed that all cytostatics led to a significant reduction in cell viability. DOX was found to be effective in both cell lines, with the reduction in cell viability being more pronounced in A673 cells than in RD-ES cells. The determined IC_50_ for doxorubicin was 8.48 × 10^−8^ M for A673 and 3.36 × 10^−7^ M for RD-ES cells.

Comparable results were shown for VIN. The IC_50_ was also lower in cell line A673 (1.21 × 10^−9^ M) than that in the RD-ES cells (6.40 × 10^−9^ M).

**Figure 1:**
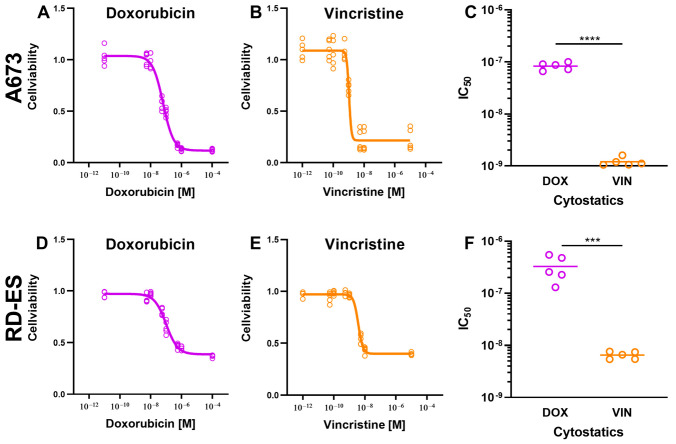
The effect of cytostatic drugs on the viability of A673 cells and RD ES cells. A total of 1 × 10^4^ cells from A673 (**A**–**C**) and RD-ES (**D**–**F**) cell lines were treated with doxorubicin (DOX) and vincristine (VIN) and incubated for 72 h. DMSO treatment was used as a control. After that, the cells were incubated with CellTiter-Blue^®^ Reagent for 2 h. The detection of cell viability was determined at the indicated time points by measuring the fluorescence signal using a multimode plate reader. The fluorescence signal of the cells treated with cytostatics was normalized to the signal of the cells treated with DMSO and presented as mean ± SD in the graphs. The graphs C and F display the IC_50_ values of the cytostatics DOX and VIN. The mean values were tested for significant differences using paired *t*-tests (**C**,**F**) and indicated as follows: *** = *p* ≤ 0.001, **** = *p* ≤ 0.0001.

Comparing the cytostatics in terms of their effectiveness showed that both cytostatics were effective, but VIN has the strongest influence on both cell lines, indicated by a significantly lower IC_50_ (Figure 1).

Analyses of growth kinetics were carried out to investigate the effects of the cytostatics on the proliferation capacity of the ES cells. To this end, the cells were incubated for 120 h, with the IC_50_ of DOX and VIN determined before. The number of living cells was determined after 4 h, 24 h, 48 h, 72 h, 96 h, and 120 h.

**Figure 2:**
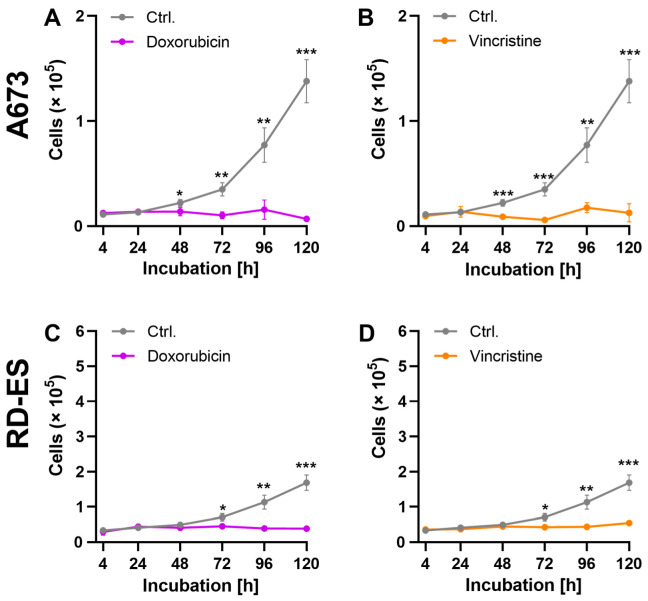
The effect of cytostatic drugs on the growth kinetics of cells: 2 × 10^4^ cells of the cell lines A673 (**A**,**B**) and RD-ES (**C**,**D**) were treated with the previously determined IC_50_ of the cytostatic drugs doxorubicin (DOX) and vincristine (VIN) and incubated for 120 h. The cells were treated with DMSO as a control. The number of living cells was determined at the indicated time points using the CASY Cell Counter and Analyzer. The graphs show the mean values ± SD. The mean values were tested for significant differences using paired *t*-tests (* = *p* ≤ 0.05, ** = *p* ≤ 0.01, *** = *p* ≤ 0.001).

The cytostatic treatment of cell line A673 showed an antiproliferative effect with all cytostatics. This effect could already be observed after 48 h of exposure to both cytostatics DOX and VIN. The cell count of the RD-ES cell line was significantly reduced by treatment with all cytostatics. A significant difference to the control was shown following treatment with DOX and VIN for 72 h (Figure 2).

### 2.2. Influence of CPP on Cell Growth

To assess the influence of CPP on cell growth, the cells were treated with CPP for 5, 10, and 20 s and incubated for 120 h. As a control, cells were treated with the carrier gas argon. The number of living cells was determined after 4 h, 24 h, 48 h, 72 h, 96 h, and 120 h.

**Figure 3:**
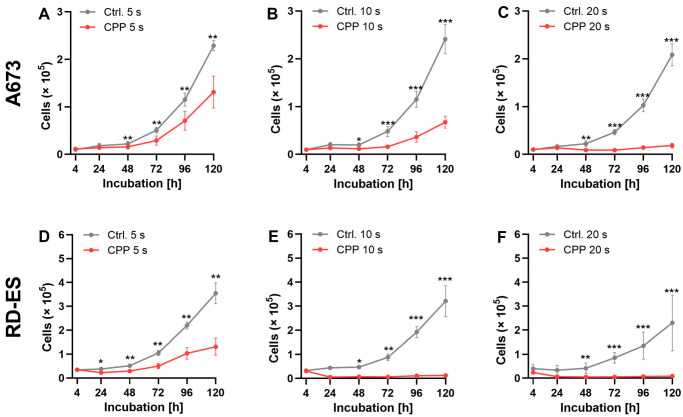
The effect of cold physical plasma (CPP) treatment on the growth progression. A total of 2 × 10^4^ cells from the A673 (**A**–**C**) and RD-ES (**D**–**F**) cell lines were exposed to CPP and argon for different time durations (5 s for (**A**,**D**), 10 s for (**B**,**E**), and 20 s for (**C**,**F**)) and incubated for 120 h. The argon treatment served as a control and was compared to the CPP treatment. The number of living cells was determined at the specified time points using CASY. The graphs depict the mean values ± SD, and the paired *t*-test was used to test for significant differences (* = *p* ≤ 0.05, ** = *p* ≤ 0.01, *** = *p* ≤ 0.001).

CPP treatment showed antiproliferative effects in both cell lines. Significant differences were evident after an incubation time of 48 h, and increased at longer incubation times. Exposure to CPP for 20 s led to a complete inhibition of cell growth (Figure 3).

### 2.3. Combination of CPP and Cytostatics

To investigate the influence of a combined treatment of CPP and a cytostatic on cell viability, A673 and RD-ES cells were treated with both CPP and a cytostatic agent and incubated for 72 h. The CPP treatment was carried out over 5, 10, and 20 s, respectively. As a control, the cells were treated with carrier gas argon and cytostatics.

The combination treatment of cell line A673 with CPP and cytostatics showed an increased reduction in cell viability compared to the control treatment with argon and cytostatics. This effect depended on the duration of the CPP treatment. For all cytostatics, treatment with CPP for 5 s had only a minor reducing effect on cell viability. A greater reduction in cell viability was observed after CPP treatment over 10 s, and over 20 s. After combined treatment, the IC_50_ of the cytostatics decreased. The reduction in the IC_50_ of the cytostatics was observed as a function of the CPP treatment duration. A longer treatment with CPP led to a more pronounced decrease in the IC_50_ of the cytostatics.

**Figure 4:**
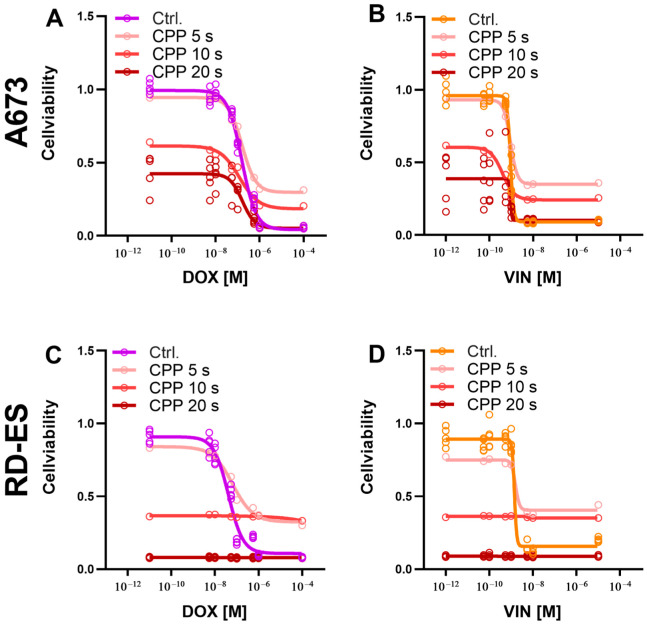
The effect of combination therapy with cytostatic drugs and cold physical plasma (CPP) on the viability of cells. Cells from the A673 (**A**,**B**) and RD-ES (**C**,**D**) cell lines were treated with the cytostatic drugs doxorubicin (DOX) and vincristine (VIN), as well as with CPP for 5 s, 10 s, and 20 s, and incubated for 72 h. The treatment with cytostatic drugs and argon served as the control. The already-determined IC_50_ values were used for the cytostatic drug treatment. Cell viability was determined using the CellTiter-Blue^®^ assay, and the fluorescence signal of the cells treated with cytostatic drugs and CPP was normalized to the signal of cells treated with cytostatic drugs and argon (control). The mean values ± SD are presented in the graph.

Compared to the control treatment with argon and cytostatics, the combination treatment with CPP and cytostatics reduced the cell viability of the RD-ES cell line. The cell viability decreased with the increasing duration of treatment with CPP. Compared to the exposure to 10 s and 20 s, CPP exposure for 5 s only slightly reduced cell viability. The 20 s CPP treatment led to an almost complete reduction in cell viability. The IC_50_ of the cytostatics was reduced depending on the duration of CPP treatment. A longer CPP treatment led to a greater reduction in the IC_50_ of the cytostatics (Figure 4).

To assess the influence of the combination therapy with the cytostatics and CPP on cell growth, cells from each cell line were treated with both CPP and cytostatics and incubated for 120 h. After 4, 24, 48, 72, 96, and 120 h, the number of living cells was determined.

**Figure 5:**
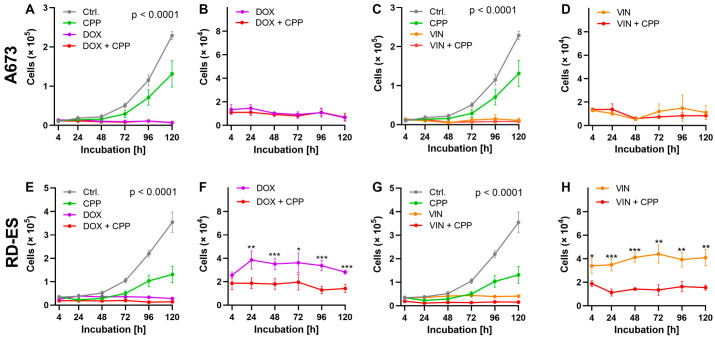
The effect of combination therapy with cytostatic drugs and cold physical plasma (CPP) on the growth kinetics of cells. The same number of cells (2 × 10^4^) from the A673 (**A**–**D**) and RD-ES (**E**–**H**) cell lines were treated in three different ways: isolated with 5 s CPP (green); isolated cells were treated with a chemotherapeutic drug—doxorubicin (DOX—purple) and vincristine (VIN—orange); combined treatment—cytostatic and CPP (red). As a control treatment argon gas was used (grey); after the treatments the cells were incubated for 120 h. The previously determined IC_50_ values were used for the chemotherapeutic treatments. with chemotherapeutic drugs alone. The number of viable cells was determined at the specified time points using the CASY Cell Counter and Analyzer. The graphs show the mean values ± SD, and two-way-repeated measure ANOVA was used. For a better understanding of the differences between the applied treatments, these were demonstrated separately with different *y*-axis scales (**B**,**D**,**F**,**H**). Tukey’s post hoc tests were applied (* = *p* ≤ 0.05, ** = *p* ≤ 0.01, *** = *p* ≤ 0.001).

The combined treatment of the cell line A673 (Figure 5A–D) showed strong antiproliferative effects, but these effects were already so strong in the isolated application of cytostatic that no statistically significant differences could be found between the two treatments (Figure 5B,D). The combined treatment of the RD-ES cell line led to a strong antiproliferative effect. The statistically significant superiority of the combined treatment of cytostatics and CPP compared to the isolated use of drugs could be shown for both DOX and VIN for this cell line (Figure 5F,H).

**Figure 6:**
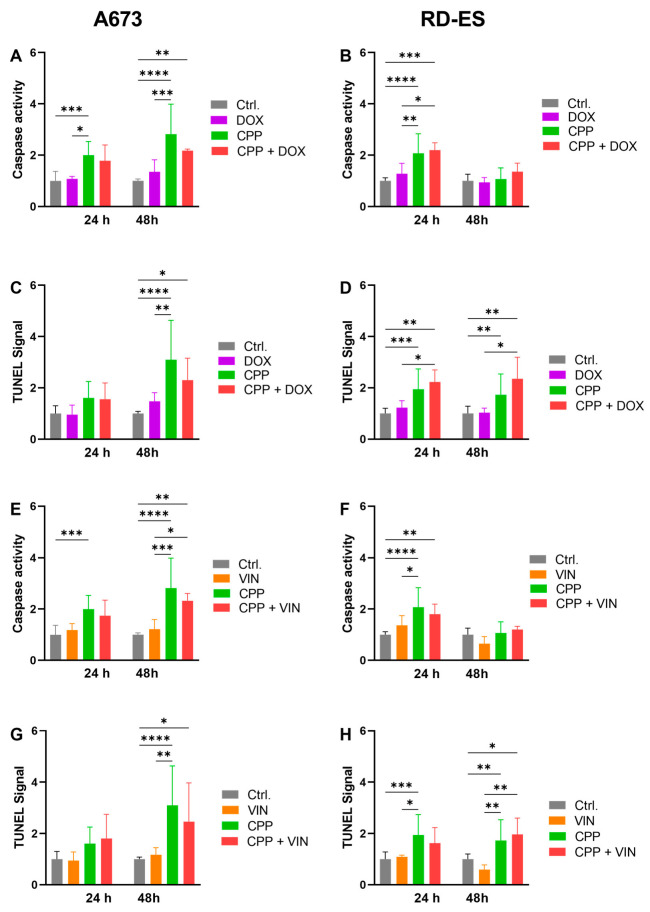
Caspase 3/7 activity assay and TUNEL assay. The cell lines A673 (**A**,**C**,**E**,**G**) and RD-ES (**B**,**D**,**F**,**H**) were treated in three different ways: isolated with 5 s cold physical plasma (CPP—green); isolated with a chemotherapeutic drug (IC_20_)—doxorubicin (DOX—purple) and vincristine (VIN—orange); combination treatment—cytostatic and CPP (red). As a control treatment, argon gas was used; after the treatments the cells were incubated. To evaluate the apoptotic effect of each therapy, and their combination, caspase 3/7 activity assay (**A**,**B**,**E**,**F**) and TUNEL assay (**C**,**D**,**G**,**H**) were used. The mean values ± SD are depicted in the graphs and were assessed for statistically significant differences using ANOVA paired *t*-tests (* = *p* ≤ 0.05, ** = *p* ≤ 0.01, *** = *p* ≤ 0.001, **** = *p* ≤ 0.0001).

To analyze the underlying mechanism of cell death, the apoptosis detection methods TUNEL and caspase 3/7 activation assay were used. The cells were isolated and treated with each: CPP for 5 s; cytostatic application at the determined IC_20_; combination of 5 s CPP prior to cytostatic (IC_20_) application. As a control, the cells were treated with argon alone. In A673 cells, CPP treatment was used alone, as the cytostatic application alone led to a significant increase in the relative caspase 3/7 compared to the control treatment (Figure 6A). The combination treatment CPP with cytostatic (both drugs) led to a significantly increased caspase 3/7 signal after 48 h treatments (Figure 6A,E). The CPP treatment of the A673 cells led to a significant increase in the relative TUNEL signal after 48 h compared to the control treatment with argon (Figure 6C). Additionally, the combination treatments (combination with each cytostatic and CPP) showed a significant increase in the relative TUNEL (Figure 6C,G).

In RD-ES, cells demonstrated a generally stronger increase in the relative caspase 3/7 signal after 24 h applying the different treatment options. After 48 h, no significant increase in the signal could be detected (Figure 6B,F). The relative TUNEL signal was increased in the RD-ES cells with different intensities after the different treatments after 24 h and after 48 h (Figure 6D,H). The TUNEL assay detected for the cell line RD-ES had a higher level of apoptotic signals with the combinations of cytostatics and CPP (both DOX and VIN) than the caspase 3/7 assay.

The comparison of the individual treatment modes with each other showed that as far as the apoptotic signals are concerned, these differed and the CPP treatment triggered these signals most strongly. These effects could be observed with both TUNEL and caspase 3/7 assay.

## 3. Discussion

Chemotherapy is the gold standard of treatment for several cancers, and most of them are routinely treated with combination chemotherapy, which has shown better outcomes than monotherapy even in incurable cancers [76]. In the case of osseous malignancies, the bony environment plays an unfavorable role, acting as a barrier hindering the diffusion of therapeutic agents [77]. Consequently, high systemic dosages are required to achieve the desired therapeutic effect [78]. However, treatment with cytostatics is also associated with various acute and late toxicities that lead to unwanted side effects such as anemia, neutropenia, nausea, vomiting, diarrhea, mucositis, nephrotoxicity, and neurotoxicity [79,80]. The specific side effects of DOX, for example, are primarily cardiotoxicity, nephrotoxicity, and liver damage [81]. It is well known that the reduction of the cytostatic dose leads to fewer side effects, but it also reduces the efficiency of the antitumoral treatment. Therefore, alternatives to a reduction of the chemotherapy dose are attractive for the clinical praxis.

After the establishment of doxorubicin as an effective therapeutic agent against Ewing’s sarcoma, this, together with cyclophosphamide, vincristine and dactinomycin, forms the basis of any chemotherapy protocol in the treatment of Ewing’s sarcoma [20,82,83,84]. Furthermore, the use of ifosfamide, alone or in combination with etoposide, has showed remarkable results in patients with Ewing’s sarcoma where standard therapies have proven to be insufficient [85,86,87,88]. Since conclusive evidence from the intergroup study conducted by the Pediatric Oncology Group and the Children’s Cancer Group showed that the addition of ifosfamide and etoposide is a useful adjunct, these have also become the gold standard of treatment for Ewing’s sarcoma [24]. In the current study, two of the most commonly used cytotoxic drugs in the treatment protocol for Ewing’s sarcoma were examined: DOX and VIN.

The role of CPP in oncology has become more popular since numerous studies showed the selective effect of this treatment on different tumors in humans [49,54,89]. Many of the anti-oncogenic mechanisms of CPP, such as apoptosis, effects on the cell membrane integrity, DNA breaks, etc., have been considered to be essential for this treatment [71,73].

In general, CPP treatment seems to be considered as a possible anti-cancer tool that supports the effects of chemotherapy. The effect of the chemotherapeutic agents unfolds through binding to DNA and the production of ROS in the cells [90]. Considering the comparable mode of action of CPP, it is assumed that a cumulative or synergistic effect of a combination of CPP and chemotherapy on cancer cells occurs, and the required effective dose of the cytostatic agent can be reduced [68]. The effect of a combination therapy of CPP and cytostatics has been described in various in vitro studies [91]. The improved efficacy of this combination therapy has been shown in some types of cancer cells, such as ovarian carcinoma [92], head and neck squamous carcinoma [93], glioblastoma [94,95,96], and melanoma [94,97,98]. In the case of melanoma, even in vivo studies have been carried out with a combination of CPP and chemotherapy, and they showed a pronounced anti-tumoral effect in reducing tumor size [98,99].

The results of the isolated application of CPP in the current study confirmed these effects. A single CPP treatment resulted in growth inhibition in all bone cancer cell lines. This inhibitory effect correlated with the CPP exposure time and was most pronounced after 20 s of treatment, leading to a complete arrest of cancer cell growth.

Previous studies have suggested that the use of CPP can lead to drug depletion [100]. Therefore, CPP mediated structural changes in the cytostatics had to be ruled out. For this purpose, the optical properties of DOX were used to perform emission spectroscopy after direct CPP treatment. It was demonstrated that CPP exposition does not cause structural changes in DOX. Consequently, the combination of CPP and cisplatin or DOX resulted in increased drug uptake in various malignancies such as melanoma, glioblastoma, pancreatic cancer, and prostate cancer [97,98,101,102]. This is further confirmation that the use of CPP and chemotherapy drugs does not have a negative impact on the effectiveness of antiproliferative drugs.

The combination of cytostatics and CPP led to an increased growth-inhibiting effect on bone cancer cells. The results showed that the treatment of the RD-ES cell line with CPP combined with DOX and VIN significantly increased the antiproliferative effect. However, these effects were not observed when utilizing the A673 cell line. This can be attributed to the fact that the sole application of chemotherapy on A673 cells resulted in an almost complete inhibition of cell growth, leaving little space for further improvement. The combination of CPP with one of the cytostatics applied on both cell lines led to reduced cell viability compared to the control treatment—a combination of argon gas and cytostatics. This effect depended on the duration of CPP treatment. A longer CPP treatment led to a higher reduction in viable bone cancer cells. In both cell lines, a 5 s CPP application was already sufficient to reduce the IC_20_ and IC_50_ of all cytostatics. These effects then became significantly more pronounced with 10 s and 20 s of CPP treatment, and the 20 s CPP treatment even led to an almost complete reduction in cell viability of the bone cancer cells. Looking at the growth kinetics of the ES cell lines after the isolated application of chemotherapy and its combination with CPP, despite the cell number at the beginning of the experiment being the same (Figure 3), there was a difference in the cell number at the initial measurement time. One of the observed effects of CPP treatments on cancer cells is that some cells die immediately as a result of the CPP application. This effect has also been observed in osteosarcoma cells treated with CPP [103]. In the current study, this explains the CPP effect of direct treatment, with the immediate death of some cells seen at the cell count determination after 4 h.

The induction of apoptosis is one of the key mechanisms associated with the anti-tumoral effect of CPP on cancer cells [104,105,106]. These CPP-induced apoptosis mechanisms have also been demonstrated for bone cancer cells such as osteosarcoma [70,107,108], Ewing’s sarcoma [71], and chondrosarcoma [72] The combination treatment with CPP and cytostatics led to varying degrees of activation of apoptosis in the individual bone cancer cell lines. The combination treatments with CPP and the various chemotherapeutic agents that triggered the increased apoptosis signals were also very heterogeneous. In cell line A673, this was observed after 48 h of combined treatment with CPP and MTX, as well as with VIN. In the RD-ES cell line, only treatment with CPP and the cytostatics MTX and DOX led to a significant increase in caspase 3/7 activity after 24 h; after 48 h, this was the case in combination with CPP and the cytostatics DOX and VIN.

In their in vitro study, Brunner et al. found similar effects of the CPP combined with low dose CIS on head and neck squamous cell carcinomas cells when evaluating the cell viability, DNA damage, and apoptosis after this treatment [93]. This supports the results of the current work by showing that the concomitant treatment of CPP and chemotherapy can enhance the therapeutic efficacy of low-dose chemotherapy drugs. Although the cellular response to CPP exposure varies for the different cell lines, a distinct response to the combination treatment of CPP and various cytostatics has been identified [109].

The effects of the combined application of CPP and chemotherapy on Ewing’s sarcoma cells in this study appear to be based on additional effects of the individual methods. The additive effect of this combination also results in a reduction in the chemotherapy dose with the same anti-cancer effect on the Ewing’s sarcoma cells. In an attempt to reduce the dose of chemotherapy and thus reduce its side effects, the combination with CPP represents an adequate option for the treatment of Ewing’s sarcoma cells.

Up to now, there have been some studies describing the in vivo application of CPP in tumor treatment [110,111]. Because CPP is a local therapy, the majority of clinical studies focus on tumors that are more superficially localized and thus more easily amenable to CPP. In the case of Ewing sarcoma, these are tumors that can only receive local treatment during surgical resection. Therefore, it is conceivable that the CPP treatment of Ewing’s sarcoma in clinical practice could be performed for the first time and once intraoperatively, with surgical removal of the tumor. The resection edges of the wound can be treated locally with CPP and used in addition to adjuvant chemotherapy. Thus, the efficiency of the antitumor treatment could be increased.

This study shows the improved anti-oncological effects of this combination, confirming the few existing studies on this topic [97,101,102]. The improved effect on ES, described for the very first time in the presented study, sheds light on novel ES treatment options.

## 4. Materials and Methods

### 4.1. Cell Culture

The Ewing sarcoma cell lines A673 (American Type Culture Collection, Manassa, VA, USA) and RD-ES (DSMZ-Deutsche Sammlung von Mikroorganismen und Zellkulturen, Braunschweig, Germany) were cultured using Dulbecco’s modified Eagle’s medium (DMEM) and Roswell Park Memorial Institute (RPMI) 1640, respectively. DMEM contained 1.0 g/L glucose, 10% fetal bovine serum, 1 mM sodium pyruvate, and 1% penicillin/streptomycin, while RPMI 1640 contained 10% fetal bovine serum and 1% penicillin/streptomycin. Both cell lines were grown in a humidified atmosphere of 5% CO_2_ at 37 °C.

### 4.2. Chemotherapeutics

In the current study, the following chemotherapeutic agents, typical for the treatment of ES, were used: doxorubicin (DOX) and vincristine (VIN) (Cayman Chemical, Ann Arbor, MI, USA). The cytostatic drugs were prepared by dissolving the dry substance in DMSO (Carl Roth, Karlsruhe, Germany). DOX was prepared at concentrations of 10^−2^ M, and VIN at concentrations of 10^−3^ M. The resulting solutions were then diluted to the desired concentrations using a dilution series in DMSO.

### 4.3. Proliferation Assay after CPP-Exposure

The Plasmajet kIN-Pen^®^ med (neoplas tools, Greifswald, Germany) was used for CPP treatment, with argon (Alphagaz 1 AIR LIQUIDE Deutschland, Düsseldorf, Germany) as the carrier gas at a flow rate of 3.5 standard liters per minute (slm). The plasma flame was guided over a 24-well plate containing 2 × 10^4^ cells per 200 µL of full medium for 5, 10, and 20 s, while a control treatment with argon was also performed. After treatment, 800 µL of the warm (37 °C) full medium was added to each well, resulting in a cell suspension of 2 × 10^4^ cells/mL. The CASY cell counter and analyzer model TT (Roche Applied Science, Mannheim, Germany) was used to determine the number of viable cells by diluting 100 µL of the cell suspension in 10 mL of CASYTon and performing three measurement cycles with a sample volume of 400 µL each.

### 4.4. Proliferation Assay after Cytostatic Exposure

To assess the potential influence of cytostatic treatment on cell growth, growth kinetics were conducted for a period of 120 h using cytostatic agents DOX and VIN. For each cell line, 2 × 10^4^ cells per 200 µL of full medium were transferred to 6 wells of a 24-well plate. Subsequently, 800 µL of warm full medium containing the corresponding cytostatic agent, dissolved at their determined IC_50_ values, was added to each well and incubated at 37 °C. If the quantification of the IC_50_ value was not possible due to limited solubility, IC_20_ values were used. As a control, a similar treatment with the carrier solution DMSO was carried out. The number of living cells was determined at 4, 24, 48, 72, 96, and 120 h using the CASY cell counter and analyzer.

### 4.5. Proliferation Assay after Cytostatic and CPP Exposure

To assess the potential impact of combining cytostatic agents with CPP on cell growth, growth kinetics were performed over a 120 h period. The cytostatic agents used were DOX and VIN, at their determined IC_50_ or IC_50_ values. CPP treatment was performed for 5 s. Each cell line was seeded with 2 × 10^4^ cells in 200 µL of full medium and directly exposed to CPP in 6 wells of a 24-well plate. After CPP exposure, 800 µL of warm full medium containing the corresponding cytostatic agent was added to each well prior to incubation at 37 °C. Control cells were treated only with cytostatic agents and incubated for 120 h. The number of live cells was determined after 4, 24, 28, 72, 96, and 120 h using the CASY cell counter and analyzer.

### 4.6. The CellTiter-Blue^®^ Cell Viability Assay after Cytostatic Exposure

The CellTiter-Blue^®^ Cell Viability Assay (Promega, Walldorf, Germany) was utilized to determine the pharmacological IC_20_ and IC_50_ of various cytostatic drugs, including DOX and VIN. In this assay, resazurin was utilized to assess the cell viability of cells after being treated with cytostatic drugs. The pharmacological IC_20_ and IC_50_ values obtained were then utilized in subsequent experiments.

Cells were first incubated for 24 h before being treated with various concentrations of the cytostatic drugs DMSO (control). The cytostatic drug preparations were performed by dissolving the dry substance in DMSO and then diluting to the desired concentrations in DMSO. The solutions were further diluted 1:100 in full medium before being administered to the cells. The cells were then incubated for 72 h at 37 °C before being treated with the CellTiter-Blue^®^ reagent.

The fluorescence signal of the cells was measured 2 h after treatment, and the formation of resorufin in living cells was detected using a multimode plate reader at 560Ex/590Em (TECAN, Männedorf, Switzerland). The fluorescence signals of the cells treated with the cytostatic drugs were then normalized to the signals of cells treated with DMSO (control) to determine their respective cell viability.

### 4.7. The CellTiter-Blue^®^ Cell Viability Assay after Cytostatic and CPP Exposure

The CellTiter-Blue^®^ Cell Viability Assay was conducted to evaluate the potential impact of a combination therapy involving cytostatic drugs and CPP on cell viability. A total of 100 µL of cell suspension containing 1 × 10^4^ cells was seeded into 96-well plates and incubated for 24 h prior to the combination therapy. CPP treatment was carried out indirectly by transferring 200 µL of the full medium into the wells of a 24-well plate and treating each well with CPP for 5 s, 10 s, and 20 s. Then, 100 µL of the treated medium was added to the cells in the 96-well plates, followed by the addition of 100 µL of warm complete medium containing the cytostatic drug, and subsequently incubated at 37 °C for 72 h. The IC_50_ or IC_20_ values of the cytostatic drugs were used for treatment. After exposure to cytostatic drugs, the cells were incubated with CellTiter-Blue^®^ reagent for 2 h. Cells exposed to argon and cytostatic drugs served as controls. The detection of cell viability was performed with fluorescence detection. The formation of fluorescent resorufins was detected using the TECAN multimode plate reader at 560Ex/590Em. The fluorescence signals of the cells treated with cytostatic drugs were normalized to the signals of the cells treated with argon and cytostatic drugs.

### 4.8. TUNEL Assay

A total of 5.0 × 10^4^ (24 h) and 2.0 × 10^4^ (48 h) A637 cells, as well as 4.0 × 10^4^ (24 h) and 2.0 × 10^4^ (48 h) RD-ES cells, were seeded into 100 µL of cell suspension in a 96-well plate. CPP treatment was performed indirectly by treating 200 µL of full medium with CPP for 5 s in a 24-well plate. Then, 100 µL of the treated medium was added to the seeded cells. Cells treated with 200 µL of the full medium treated with argon for 5 s served as controls. After the CPP treatment, the cells were treated with DOX and VIN at their respective IC_20_ concentrations, and control with argon and the cytostatic drugs was also performed. Controls with untreated cells (1 negative and 1 positive; nuclease treated) were included on each plate. A corresponding second plate was treated in parallel to normalize the absorption values to cell numbers. The TUNEL assay (R&D Systems, Minneapolis, MN, USA) was performed 24 h or 48 h after treatment according to the manufacturer’s protocol using the TECAN multimode plate reader. The relative TUNEL signals of cells treated with CPP, cytostatic or combination therapy were normalized to the mean relative TUNEL signals of cells treated with argon (control).

### 4.9. Caspase Assay

The CellEvent^TM^ Caspase 3/7 green Detection Reagent (Thermo Fisher Scientific, Waltham, MA, USA) was used to detect apoptosis by performing the Caspase 3/7 assay. The detection reagent binds fluorescently to DNA, and this binding is inhibited by the DEVD peptide. Upon activation of Caspases 3 and 7, the peptide is cleaved, allowing the binding to occur. The treatment was performed similarly to the TUNEL assay.

After the incubation period (24 h and 48 h), the medium was carefully aspirated and 100 µL of Caspase 3/7 detection solution was added to the wells of the 96-well plate. The plate was then incubated for 45 min at 37 °C.

Following the 45-min incubation, fluorescence was measured using the TECAN multimode plate reader at 495Ex/535Em. The absorption per cell was calculated using the determined cell numbers of the parallel plate. The relative Caspase 3/7 signals of cells treated with CPP, cytostatic or combination therapy were normalized to the mean relative Caspase 3/7 signals of cells treated with argon (control).

### 4.10. Statistics

For data analysis and visualization, GraphPad Prism Version 9.1.2 (GraphPad Software Inc., La Jolla, CA, USA) was used. The results of *p* ≤ 0.05 of at least three independent experiments were considered significant and data were given as the mean ± SD. Differences were examined using an ANOVA test or *t*-test, as indicated in the figure captions.

## 5. Conclusions

In this study, it was shown for the first time that the combination of CPP with various cytostatics might be a favorable therapeutic approach for rare bone tumors. The CPP treatment itself has no harmful effect on the drug, but rather enhanced its inhibitory effect on cell proliferation and cell viability and promoted apoptosis of Ewing’s sarcoma cell lines. These results, obtained in vitro, represent a promising first step towards novel concepts to treat Ewing’s sarcomas and related metastases.

## Data Availability

The data that support the findings of this study are available from the corresponding author upon reasonable request.
